# Identification of immunodominant IgE binding epitopes of Der p 24, a major allergen of *Dermatophagoides pteronyssinus*

**DOI:** 10.1186/s13601-019-0266-7

**Published:** 2019-05-23

**Authors:** Ze-Lang Cai, Jia-Jie Chen, Zhen Zhang, Yi-Bo Hou, Yong-shen He, Jin-Lyu Sun, Kunmei Ji

**Affiliations:** 1 Department of Biochemistry and Molecular Biology, Health Science Center of Shenzhen University, Shenzhen, China; 2 Department of Allergy of Peking Union Medical College Hospital, Peking Union Medical College, Chinese Academy of Medical Sciences, Beijing Key Laboratory of Precision Medicine for Diagnosis and Treatment on Allergic Diseases, Beijing, China

**Keywords:** House dust mite, Der p 24, Immunodominant IgE epitope, IgE-binding

## Abstract

**Background:**

The identification of house dust mite (HDM) allergens and epitopes is important for allergy diagnosis and treatment. We sought to identify the *Dermatophagoides pteronyssinus* group 24 allergen (Der p 24) and to identify its immunodominant IgE epitope(s).

**Methods:**

Der p 24 cDNA was cloned and expressed in a pET expression system. The IgE binding activity of purified recombinant (r)Der p 24 was evaluated by western blotting. Truncated Der p 24 proteins and overlapping synthetic polypeptides were subjected to IgE binding assays. Balb/c mice were immunized to investigate IgE epitope induction of IgE production. IgE binding of the 32 N-terminal residues of Der p 24 was compared to other Der p epitopes in enzyme-linked immunosorbent assays and dot blot assays. Human skin prick tests (SPTs) were performed.

**Results:**

We cloned and expressed Der p 24 cDNA (GenBank accession no. KP893174.1). HDM allergic sera bound rDer p 24 in vitro and 5/10 HDM allergic patients (50%) had positive SPT reactions to rDer p 24. The immunodominant IgE epitope of Der p 24 was localized to the N-terminal 32-residue region, which produced a high specific IgE antibody titer in vivo and promoted mast cell β-hexosaminidase release. The IgE binding activity this N-terminal epitope of Der p 24 was stronger than that of Der p 1 or Der p 2 IgE epitopes.

**Conclusions:**

We identified Der p 24 as a major HDM allergen with strong IgE binding activity via an immunodominant IgE epitope in the N-terminal 32-residue region, which triggers IgE production in vivo. The identified Der p 24 epitope may support HDM allergy diagnosis and treatment.

## Introduction

Allergic diseases constitute the most widespread type of immune disorder in the world [1]. The house dust mite (HDM) species *Dermatophagoides pteronyssinus* (Der p) and *Dermatophagoides farinae* (Der f) are major sources of indoor inhaled allergens underlying immunoglobulin E (IgE)-mediated anaphylactic reactions [2], which can manifest as allergic asthma, allergic rhinitis, and allergic dermatitis [3]. HDM allergens, which trigger reactions in half of allergy sufferers [4], are important components of allergic disease diagnosis and treatment [5]. The rate of HDM allergy is high in developed countries [6]. The previous clinical studies showed that HDM allergic sensitization is above 20% in Europe [7] and up to 40% in North America [8]. Thus, HDM triggered allergy should be a common event.

The identification of novel allergens benefits allergic disease diagnosis and treatment. Of the 38 HDM allergen groups identified, allergens from 32 groups and 23 groups have been identified for Der f and Der p, respectively, and catalogued in the World Health Organization and International Union of Immunological Societies (WHO/IUIS) allergen database (http://www.allergen.org). The most clinically important HDM allergens identified thus far have been group 1, group 2, and group 23 allergens [9], with recent evidence suggesting that HDM group 24 allergens may also be clinically important [10]. Previously, we found that a group 24 Der f allergen, namely Der f 24, was a ubiquinol cytochrome C reductase binding (UQCRB) protein homolog [10]. UQCRB proteins function to maintain complex III in the electron transport chain across phylogenetically diverse species, including insects and mammals [11]. We found that Der f 24 protein yields strong IgE reactivity with sera from HDM allergic patients both in vitro and in vivo [10]. Despite the substantial genetic and therefore protein similarities between Der p and Der f mites, the two species have been shown to have divergent allergen components [12]. Thus, it remains to be determined whether there is a Der p UQCRB protein homolog, which would constitute a novel HDM allergen.

Allergenicity depends upon IgE binding activity. IgE epitopes, also known as B cell epitopes, are the sites where serum IgEs bind allergens to induce inflammatory reactions [13, 14]. Allergen IgE epitope analysis results can provide a good indicator of patients’ allergy sensitivities and help to predict clinical severity and tolerance development potential [15]. Thus far, B cell epitopes have been identified for only group 1, 2, 3, 7, 11, 13, 23 and 33 HDM allergens [16–21]. Traditionally, allergen IgE epitopes have been assumed to be involved in the native functions of allergen proteins [22]. However, it is also possible that allergenic IgE epitopes may be a simple consequence of the local amino acid (aa) sequence or produced in relation to its protein function.

Demonstration of the IgE epitope of a Der p 24 allergen would be helpful for advancing HDM allergy diagnosis and treatment. The aims of this study were firstly to examine whether a putative novel Der p 24 allergen can be identified, and secondly, if so, to determine its immunodominant IgE epitope. To achieve these aims, we performed IgE binding activity experiments, employing western blot, dot blot, and enzyme-linked immunosorbent assay (ELISA) in vitro methods as well as in vivo mouse immunization and human skin prick tests (SPTs), and then compared IgE binding activity across Der p groups’ IgE epitopes.

## Materials and methods

### Serum samples

Serum samples from 50 HDM allergic patients (23 males and 27 females; age range, 18–55 years) and 20 non-allergic control group individuals (8 males and 12 females; age range, 18–55 years) were provided by the First Affiliated Hospital of Guangzhou Medical College. The HDM allergic group consisted of patients who had experienced HDM-triggered anaphylaxis or IgE levels higher than 3 when measured by the ImmunoCAP allergen detection system (Pharmacia Diagnostics, Uppsala, Sweden). A subsample of 10 allergic patients were recruited separately for Der p extract and recombinant (r)Der p 24 skin prick testing (demographic and clinical characteristics summarized in Table 1). Ethics approval was obtained from the First Affiliated Hospital of Guangzhou Medical College. Permission to conduct this study was obtained from the Ethics Committee of the First Affiliated Hospital of Guangzhou Medical College (No. 2012-51). Informed consent was obtained from all individual participants included in the study. All procedures involving human participants were in accordance with the ethical standards of the committee.

**Table 1:** Skin reactivity

Patient	Sex/age, y	Clinical history	SPTs^a^	
			Der p extract	rDer p 24
1	Female/39	AR + BA	3 +	2 +
2	Female/25	AR	3 +	–
3	Female/27	BA	3 +	–
4	Male/52	AR	4 +	2 +
5	Male/38	BA	3 +	–
6	Male/24	AR	4 +	2 +
7	Female/40	AR	3 +	–
8	Female/23	AR	3 +	–
9	Male/44	AR + BA	3 +	2 +
10	Female/39	AR	3 +	2 +

Skin reactivity to Der p extract and rDer p24 (10 μg/ml) with normal saline as a negative control and histamine as a positive control. Negative results were obtained from 12 healthy control subjects

*AR* allergic rhinitis, *BA* bronchial asthma

^a^Skin reaction ≥ 2 + considered positive; − negative result

### Cell culture

Rat basophilic leukemia cell lines (RBL-2H3 cells) were purchased from the Chinese Academy of Sciences Cell Bank. The cells were cultured in Dulbecco’s Modified Eagle Medium supplemented with 10% fetal bovine serum and 1% l-glutamine–penicillin–streptomycin in 5% CO_2_ incubator at 37 °C. Cells were split every 2–3 days with 0.25% (wt/v) trypsin/1 mM EDTA (Thermo Fisher Scientific).

### Cloning, expression, and purification of rDer p 24 and its truncated form proteins

The Basic Local Alignment Search Tool (BLAST) was used to find a UQCRB protein homolog in Der p based on aa sequence. The Der p UQCRB protein was expressed as inclusion bodies, solubilized with guanidine hydrochloride, purified with Ni-NTA resin, and analyzed by 12% sodium dodecyl sulfate polyacrylamide gel electrophoresis (SDS-PAGE). Specific truncated forms of Der p 24 were used to screen for IgE epitopes. Der p 24 open reading frame cDNA was amplified by polymerase chain reaction (PCR). The sequences of the primers used in PCRs and expression subcloning are shown in Table 2. Relative cDNA sequences encoding Der p 24 or its truncated-form proteins were synthesized by Genescript Corporation (Nanjing, China). The truncated forms of Der p 24 employed, called Der p 24 (1–98aa), Der p 24 (1–78aa), Der p 24 (1–58aa), Der p 24 (33–118aa), and Der p 24 (21–118aa), are shown in Fig. 2a.

**Table 2:** Forward (F) and reverse (R) primers used in PCRs and expression subcloning

Primer	Sequence^a^
Der p 24-F	5′-GGATCCATGGTTCATCTTACAA-3′
Der p 24-R	5′-AAGCTTCAATCTTTCGACAAG -3′
Der p 24 (1–98aa)-F	5′-GGATCCATGGTTCATCTTACAA-3′
Der p 24 (1–98aa)-R	5′-AAGCTTCAAAGATATGGTGTA-3′
Der p 24 (1–78aa)-F	5′-GGATCCATGGTTCATCTTACAA-3′
Der p 24 (1–78aa)-R	5′-AAGCTTCATTTCGGTAAAAAT-3′
Der p 24 (1–58aa)-F	5′-GGATCCATGGTTCATCTTACAA-3′
Der p 24 (1–58aa)-R	5′-AAGCTTCATTGATCATAAAGA-3′
Der p 24 (33–118aa)-F	5′-GGATCCTACACTGATCCAGCTC-3′
Der p 24 (33–118aa)-R	5′-AAGCTTCAATCTTTCGACAAG-3′
Der p 24 (21–118aa)-F	5′-GGATCCTACGGTCTTCAAGGAT-3′
Der p 24 (21–118aa)-R	5′-AAGCTTCAATCTTTCGACAAG-3′

^a^Restriction enzyme sites are underlined: *BamH I*, GGATCC; *Hind III*, AAGCTT

PCR products were confirmed by DNA sequencing, cloned into pET-His vectors, and transformed into *Escherichia coli* BL21 (DE3) plysS for recombinant protein expression. The expression and purification of recombinant proteins was performed as described previously [23]. Following Ni-NTA gel affinity chromatography purification, the expressed proteins were used in IgE binding activity experiments. Protein concentration was measured by the Bradford method using bovine serum albumin (BSA) as a standard. Purified Der p24 protein was analyzed by SDS-to detection of peptide coverage using mass spectrometry (MS) (Beijing Baitai Parker, Beijing, China).

### Artificial synthesis of overlapping polypeptides

Overlapping polypeptides of Der p 24 were designed based on the N-terminal 32 residues region of Der p 24, as shown in Fig. 3a. These polypeptides were conjugated with BSA protein, producing BSA-Der p 24(1–32aa), BSA-Der p 24(1–20aa), BSA-Der p 24(10–23aa), BSA-Der p 24(13–26aa), BSA-Der p 24(16–29aa), and BSA-Der p 24(19–32aa), respectively. The main linear IgE epitopes identified previously for Der p 1 (^81^EYIQHNGVVQESYY^94^ and ^101^QSCRRPNAQRF^111^) and for Der p 2 (^65^VPGIDPNACHYMK^78^) were used as positive IgE binding controls. All of these polypeptides were synthesized by Shanghai Qiang Yao Biotech (Shanghai, China) and coupled with BSA at the N-terminus or C-terminus with a purity > 95%; the sequences were confirmed by matrix-assisted laser desorption ionization time-of-flight MS analysis.

### IgE western and dot blotting

Purified proteins were separated by 12% SDS-PAGE and then transferred to polyvinylidene-fluoride membranes (Millipore, USA) for western blotting. For dot blotting, 2-µg aliquots of polypeptides were applied serially onto nitrocellulose membrane (Millipore, USA); BSA was used as a control. The membranes were blocked with 5% Difco™ skim milk (DSM, BD Biosciences, USA) diluted in Tris buffered saline containing 0.05% Tween 20 (TBST) at 4 °C overnight. Serum samples were incubated for 2.5 h at 37 °C, and then incubated with horse radish peroxidase-conjugated mouse anti-human IgE antibody (1:2000 in TBST with 1% DSM) for 1 h at 37 °C. Antigen–antibody complexes on membranes were visualized with a Pierce™ 3′-diaminobenzidine substrate kit.

### IgE-ELISA

Microtiter plates were coated with 100 μl of 2 μg/ml polypeptides (diluted in carbonate buffer, pH 9.6) at 4 °C overnight. The plates were blocked with 5% (w/v) DSM in phosphate-buffered saline containing 0.05% Tween 20 for 3 h at 37 °C. After washing, the plates were incubated with serum from HDM-allergic patients (1:10) for 2.5 h at 37 °C, followed by mouse anti-human IgE-horse radish peroxidase conjugated (1:2000) for 1 h at 37 °C. The signal was detected by adding 3,3′,5,5′-tetramethylbenzidine substrate and the reaction was stopped with 2 M H_2_SO_4_. Absorbance was measured at 450 nm by a microplate reader (Bio-Rad, USA). IgE-ELISA results with a positive/negative value > 2.1 were considered positive (P/N, ratio of optical density values of positive and negative result samples). All tests were performed in triplicate.

### Skin prick tests

The Skin prick test (SPT) was performed as previously described [24]. Specifically, ethics approval was obtained from the First Affiliated Hospital of Guangzhou Medical College (No. 2012-51). All participants agreed to participate in this study voluntarily and gave written informed consent. Both the patients and healthy controls confirmed that they had not been injected with any drugs or took any medicine during 12 h before the SPT. SPT was performed with recombinant Der p24 protein (10 μg/ml in PBS) and standardized *D. pteronyssinus* extracts (ALK Abello, Denmark) in 10 dust mite-allergic patients with allergic rhinitis and/or asthma and 12 healthy controls (Table 1). The result was defined as positive when the prick spot became a raised, itchy wheal surrounded by a reddened flare; otherwise, the result was considered negative in the absence of these signs. Negative results were obtained from 12 healthy control subjects.

### Structure modeling of Der p 24

Online SWISS-MODEL software was used to model the three-dimensional structure of Der p 24 based on its aa sequence. The 32 N-terminal residues were labelled with a red mark.

### Sensitized mice

Balb/c female mice (6–8 weeks old), purchased from Guangzhou Experimental Animal Center (Guangdong, China), were maintained under specific pathogen free conditions. Mice were sensitized by intraperitoneal injection of 50 μg of OVA or OVA-Der p24(1–32aa) antigen with 2 mg of Adjuvant (Imject™ Alum Adjuvant, Thermo Fisher Scientific Inc., USA) on experimental days 0, 7, and 14. They received three OVA or OVA-1–32aa antigen boosters by subcutanoeous injection on experimental days 25, 26, and 27. On day 28, serum levels of IgE and IgG specific to BSA-Der p24(1–32aa) were measured by ELISA as described previously [25]. Sera from these mice were later subjected to IgE-western blot analysis and a mast cell degranulation assay wherein ß-hexosaminidase release by RBL-2H3 cells was assessed (described below). Non-sensitized control mice were administered phosphate buffered saline (PBS) according to the above treatment schedule.

### RBL-2H3 degranulation assay

Degranulation assays were performed as previously reported with slight modifications [26]. Briefly, RBL-2H3 cells were cultured as described in the above cell culture section. Cells were seeded in 96-well plates for 18 h. After being washed three times with Tyrode’s buffer (25 mM PIPES, 125 mM NaCl, 2.7 mM KCl, 5.6 mM glucose, 1 mM CaCl_2_, and 0.1% BSA, pH 7.4), cells were exposed to sera of sensitized mice for 2 h and then stimulated with 10 μg/ml BSA-1–32aa conjugate for 45 min. Degranulation was assessed by measurement of ß-hexosaminidase release into the supernatant and of unreleased enzyme in the respective cell lysate. The percentage release of total ß-hexosaminidase content was calculated from these values and reported.

### Histologic analysis

Lung tissues were removed under aseptic conditions for hematoxylin and eosin (H&E) staining. The tissues were fixed in 4% paraformaldehyde for 72 h, embedded in paraffin. The paraffinized samples were sliced into 5-μm sections, stained with H&E, and examined under 100 × magnification. Peribronchial and perivascular inflammation were assessed.

### Statistical analysis

Data are presented as means with standard deviations (SDs). Statistical analyses were conducted in GraphPad Prism7 (GraphPad Software, La Jolla, Calif). Differences between groups were determined by analysis of one-way variance (ANOVA) followed by Dunnett’s *t* test for multiple comparisons. P values < 0.05 were considered significant.

## Results

### Identification of Der p 24 as a novel HDM allergen

**Fig. 1:**
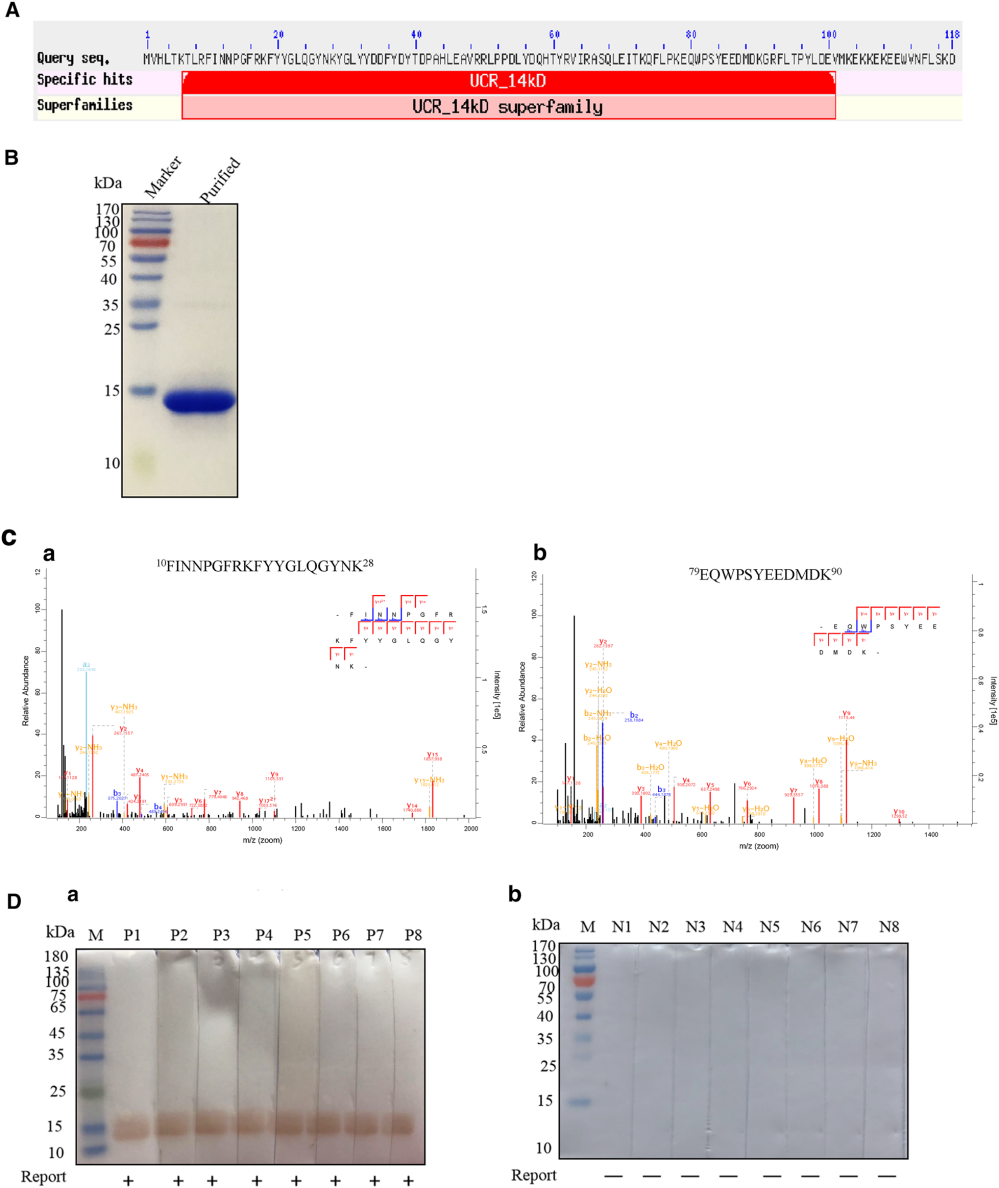
Cloning, recombinant expression, and allergenic identification of Der p 24. **A** Sequence analysis of Der p 24 protein. BLAST homology comparison of Der p 24 and Der f 24 (GenBank No. AJK91617.1). After codon optimization, synthesized Der p 24 cDNA was subcloned into pET expression system to generate Der p 24 (theoretical molecular mass = 14 kDa; GenBank accession no. KP893174.1). **B** SDS-PAGE analysis of purified rDer p 24 stained with Coomassie brilliant blue. **C** Peptide coverage of rDer p 24 based on MS analysis: 10–28aa peptide data shown in **a** and 79–90aa peptide data shown in **b**. **D** IgE binding activity determined by IgE-western blots of rDer p 24 with individual sera from 8 HDM-allergic patients (**a**) and 8 healthy non-allergic individuals (**b**)

BLAST analysis revealed a UQCRB protein homolog in Der p (Fig. 1A). The codon optimized cDNA sequence of this Der p UQCRB protein was cloned into pET expression vector and the resultant purified product was identified by DNA sequencing and found to have a theoretical molecular mass of 14 kDa (Fig. 1B; GenBank no. KP893174.1). MS analysis indicated that a 97.5% coverage rate was achieved. Representative results from two coverage peptides are shown in Fig. 1C. These data confirm that a rDer p UQCRB protein homolog was obtained.

IgE western blot analysis showed that the rDer p UQCRB protein reacted with serum IgEs from individual HDM allergic patients (Fig. 1D). In SPTs with the Der p UQCRB protein, 5/10 allergic patients (50%) had positive reactions (Table 1). Based on the present data, the WHO/IUIS allergen nomenclature subcommittee published this allergenic protein as Der p 24 (http://www.allergen.org/viewallergen.php?aid=855).

### Localization of IgE epitope of Der p 24 to N-terminal region

**Fig. 2:**
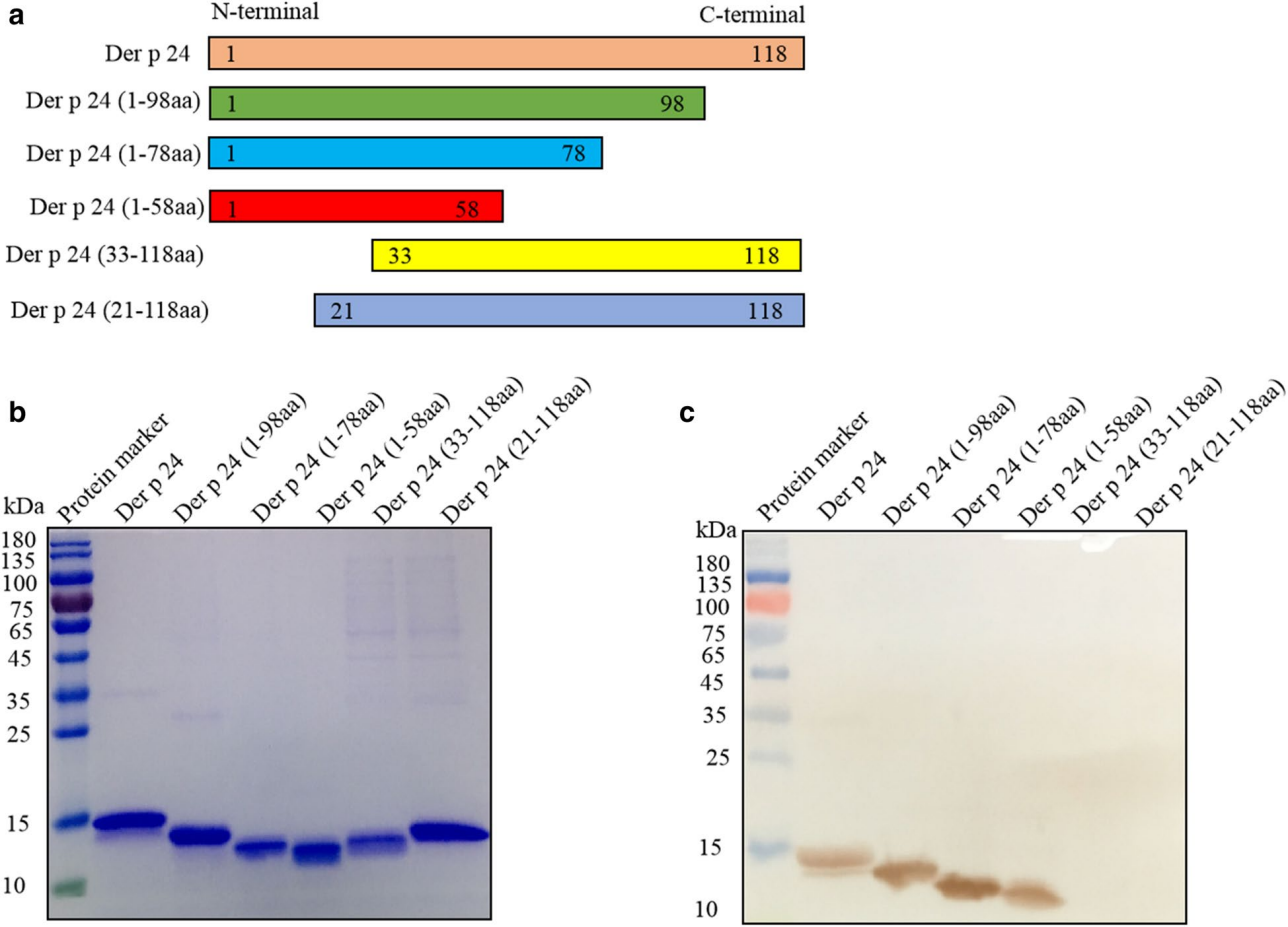
Localization of an IgE epitope of Der p 24 to the N-terminus. **a** Schematic representation of truncated Der p 24 fragments used for IgE epitope mapping. **b** SDS-PAGE analysis of rDer p 24 and five truncated forms of rDer p 24 after Ni-NTA resin purification. The gel was stained with Coomassie Brilliant Blue. **c** IgE binding activity of truncated Der p 24 fragments determined by IgE-western blots with pooled sera from 8 HDM-allergic patients

Truncated forms of Der p 24 used to screen IgE binding epitopes were expressed in a pET expression system, purified with Ni-NTA resins, and analyzed by SDS-PAGE (Fig. 2a, b). Western blot analysis showed strong IgE reactivity of three of the truncated proteins, namely Der p 24 (1–98aa), Der p 24 (1–78aa), and Der p 24 (1–58aa), with serum from HDM allergic patients. The two truncated proteins that were unreactive, Der p 24 (33–118aa) or Der p 24 (21–118aa), lacked the N-terminal region of Der p 24 (Fig. 2c).

### Immunodominant IgE epitope in 32 N-terminal residues of Der p 24

**Fig. 3:**
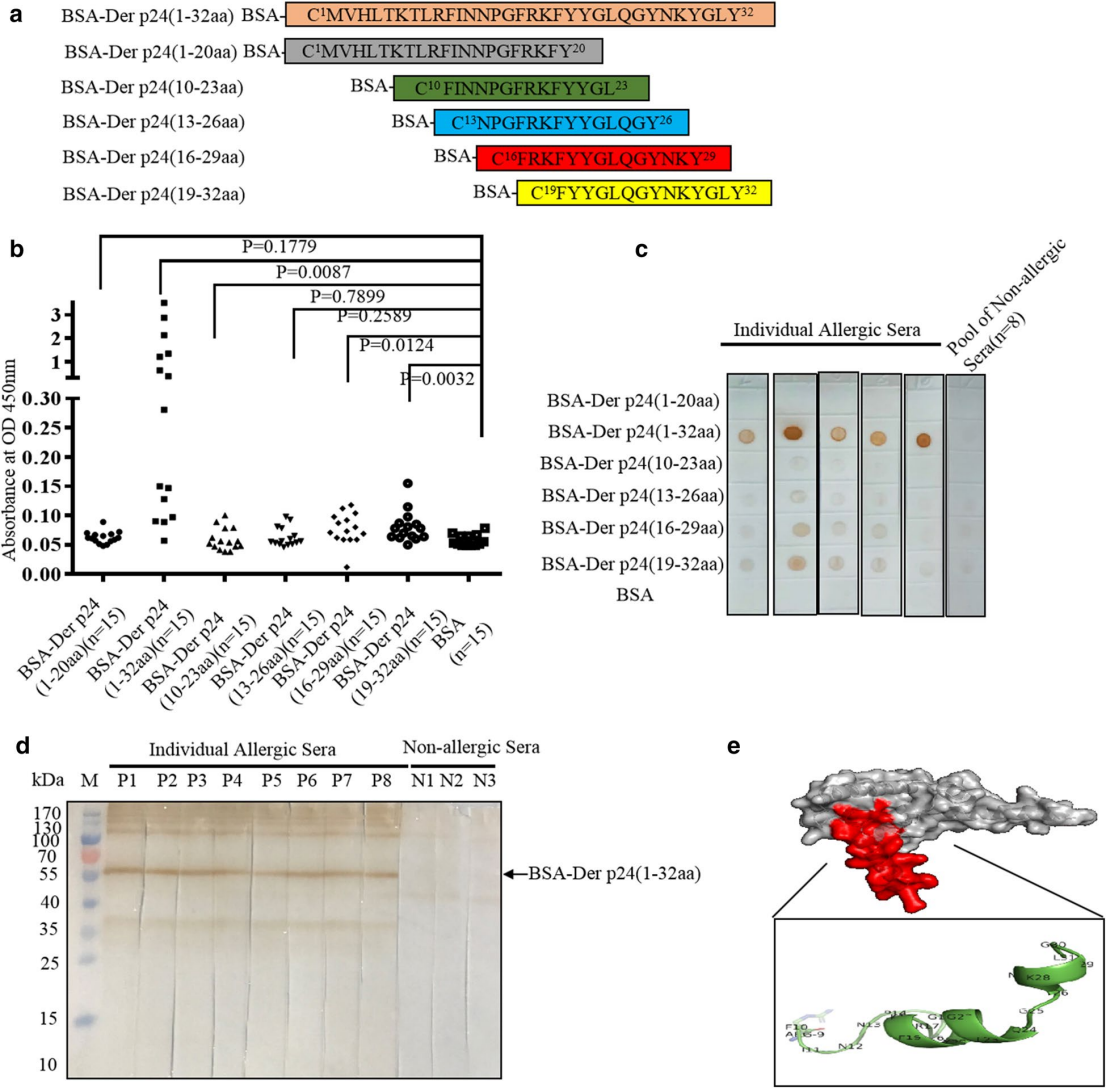
Identification of N-terminal 32 residues of Der p 24 is an immunodominant IgE epitope. **a** Synthesized overlapping polypeptides designed according to the N-terminal 32 residue sequences and conjugated with BSA protein for comparative analysis of IgE binding reactivity. **b** IgE-ELISAs of overlapping polypeptides with individual sera from 15 HDM-allergic patients. P values < 0.05 were considered significant. **c** IgE-dot blot of overlapping polypeptides (2 μg each) incubated with sera from 5 HDM-allergic patients (P1–P5; subset from experiment in B with positive reactions) and pooled sera from 3 non-allergic controls (N). **d** IgE-western blot of BSA-Der p 24(1–32aa) polypeptide conjugate with serum samples from 8 HDM-sensitized patients (P1–P8) and 3 non-allergic individuals (N1–N3). **e** Homology-based three-dimensional model of the structure of Der p 24, which included two alpha helices and two random coils. The N-terminal-32 residue region is labelled red

The IgE binding activity of synthetic overlapped polypeptides coupled with BSA, namely BSA-Der p 24(1–32aa), BSA-Der p 24(1–20aa), BSA-Der p 24(10–23aa), BSA-Der p 24(13–26aa), BSA-Der p 24(16–29aa), and BSA-Der p 24(19–32aa) (Fig. 3a), was evaluated with IgE-ELISAs (Fig. 3b), IgE-dot blots (Fig. 3c), and IgE-western blots (Fig. 3d). The BSA-Der p 24(1–32aa) conjugate produced a stronger positive reaction with serum IgEs from individual HDM-allergic patients than did the other conjugates (Fig. 3b, c). BSA-Der p 24(16–29aa) and BSA-Der p 24(19–32aa) were bound weakly by IgEs in HDM allergic sera (Fig. 3c). The N-terminal region of Der p 24 exhibited multi-immunodominant epitopes. IgE-western blotting showed that BSA-Der p 24(1–32aa) was bound by serum IgEs from eight HDM allergic patients but not bound by serum IgEs from three non-allergic individuals (Fig. 3d).

Homologous three-dimensional structural modeling of Der p 24 in SWISS-MODEL software indicated that the 32-residue N-terminal region has three ɑ-helices and a free loop structure positioned on the outside of whole folded protein (Fig. 3e). This external location may facilitate IgE binding. These results indicate that the N-terminal 32 residues of Der p 24 form an immunodominant complex with multi-type B cell epitopes, including conformational and linear epitopes, of which a conformational epitope is dominant.

### N-terminal region of Der p 24 induces IgE production in vivo

**Fig. 4:**
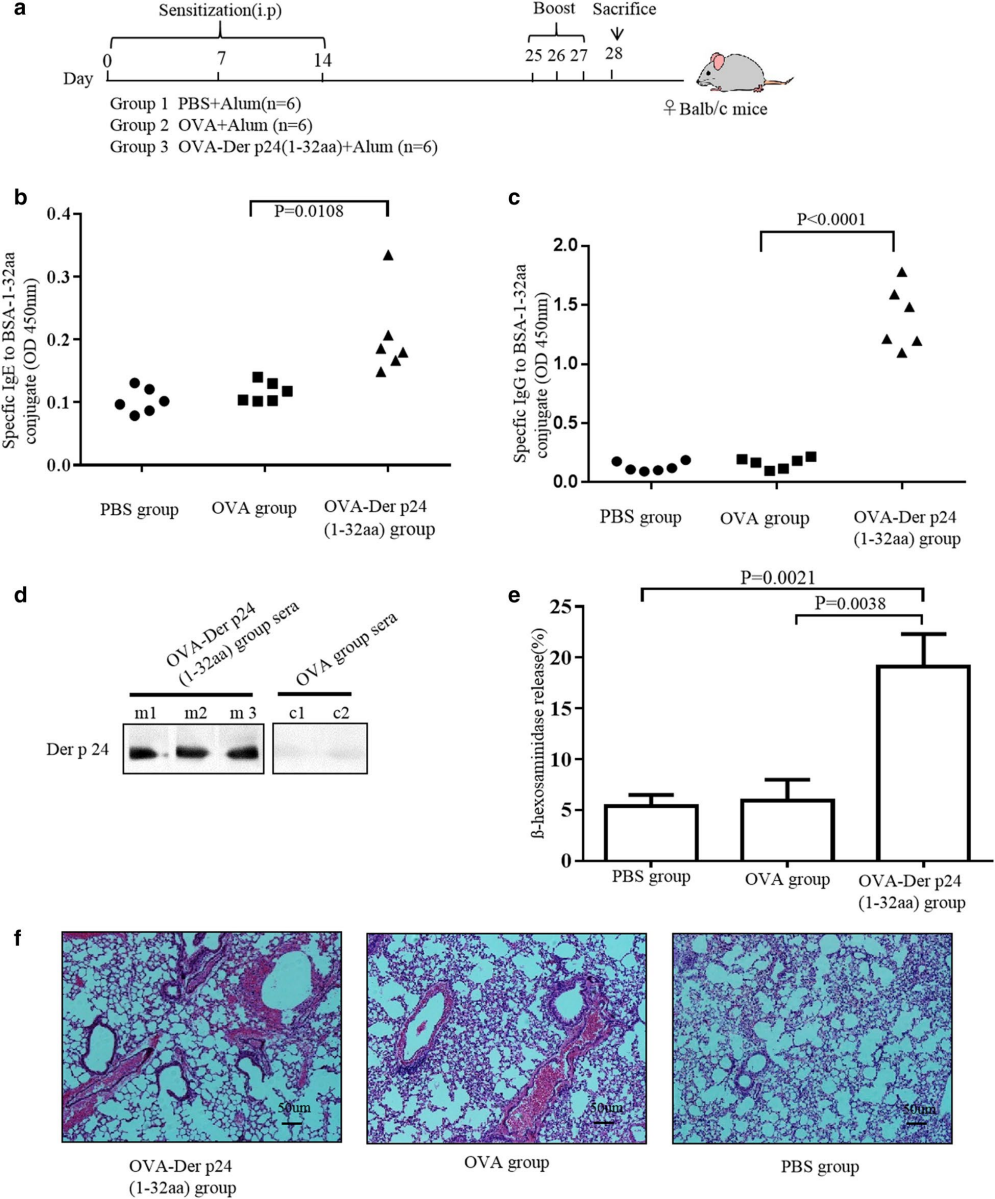
Induction of IgE production by N-terminal 32-residue region of Der p 24 in Balb/c mice. **a** Experimental design. Postmmunization indirect ELISA detection of specific IgG (**b**) and IgE (**c**) antibodies against the conjugate. **d** IgE-western blot of rDer p 24 with sera from the OVA-Der p 24(1–32aa) (m1–m3 represent three different individuals) and OVA (c1 and c2 represent two different individuals) groups. **e** Significantly greater RBL-2H3 cell ß-hexosaminidase release was induced by sera from the OVA-Der p24(1–32aa) group than that induced by sera from the OVA and PBS groups. **f** Light photomicrographs of H&E-stained sections of lung tissues in mice challenged with OVA-Der p24(1–32aa), OVA, or PBS (× 100 magnification). P values < 0.05 were considered significant

Balb/c mice were immunized with an OVA-Der p 24(1–32aa)-conjugate, OVA, or PBS (negative control) in accordance with the experimental design shown in Fig. 4a. Subsequently, the OVA-Der p 24(1–32aa) group produced high specific IgG and IgE titers against the BSA-Der p 24(1–32aa) conjugate (Fig. 4b, c). Serum from mice immunized with OVA-Der p 24(1–32aa), but not OVA- or PBS-injected mice, showed specific IgE binding to Der p 24(1–32aa) (Fig. 4d) and promoted significant release of β-hexosaminidase from mast cell line RBL-2H3 mast cells with the addition of BSA-Der p 24 (1–32aa) conjugate. The mean ß-hexosaminidase release observed with the OVA-Der p 24(1–32aa) group was 19.14%, consistent with mast cell degranulation, whereas the percentages obtained for the OVA and PBS groups were just 5.95% and 5.38%, respectively (Fig. 4e). In addition, H&E histology showed that lungs from mice in the OVA-Der p 24(1–32aa) group exhibited enhanced signs of inflammation compared to the PBS group (Fig. 4f). These results demonstrate that the immunodominant IgE epitopes of Der p 24 identified in this study can induce specific IgE production in vivo.

### High IgE binding of N-terminal epitope of Der p 24

**Fig. 5:**
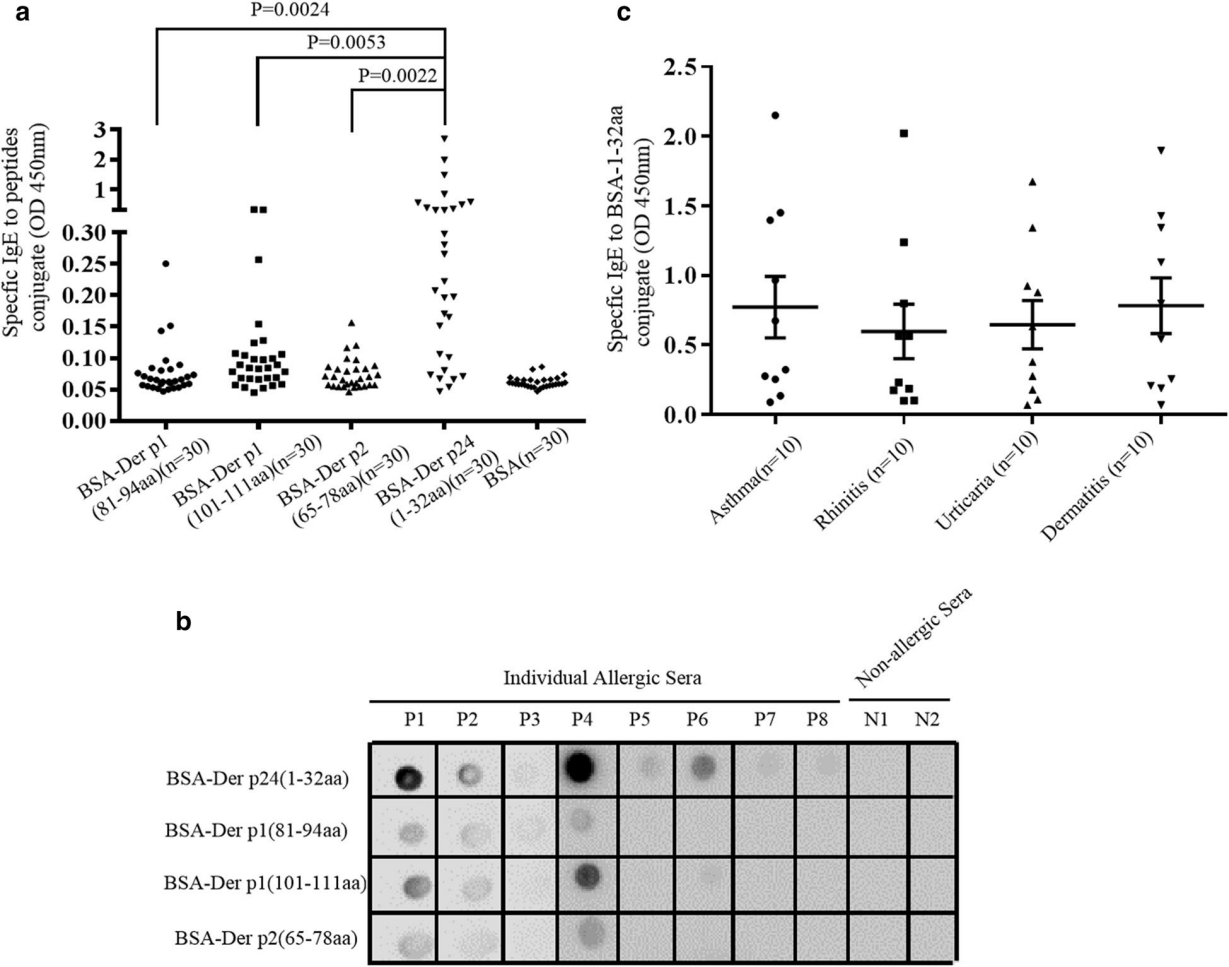
IgE binding activity of N-terminal epitope of Der p 24 and main linear IgE epitopes of Der p 1 or Der p 2. **a** Indirect IgE-ELISA with serum samples from HDM-sensitized patients (N = 30) and non-allergic controls (N = 2). **b** IgE-dot blot analysis with sera from HDM allergic patients (N = 8, P1–P8) and non-allergic individuals (N = 2, N1–N2). **c** Indirect IgE-ELISA for detecting specific IgE against immunodominant IgE epitopes of Der p 24 with individual serum samples from HDM allergic patients with symptom manifestations of only asthma, rhinitis, urticaria, or dermatitis (N = 10 per manifestation group). Mean values are shown with standard errors and p values < 0.05 were considered significant

IgE-ELISAs with separate serum samples from 30 individual HDM allergic patients showed strong IgE binding activity of the 32-residue N-terminal region of Der p 24, exceeding that seen for the main linear IgE epitopes of Der p 1 or Der p 2 (Fig. 5a). IgE-dot blot results obtained for BSA-Der p 24(1–32aa) from Der p 24 and similarly constructed Der p 1 and Der p 2 peptide region conjugates with sera from eight HDM allergic patients and two non-allergic control individuals (randomly selected from allergic and control groups) are shown in Fig. 5b. IgE-ELISAs of serum samples from patients with asthma, rhinitis, urticarial, and dermatitis allergy manifestations (N = 10 per manifestation) revealed no correlation between the IgE binding activity of Peptide-1–32aa from Der p 24 and allergic disease symptoms (Fig. 5c).

## Discussion

Based on the presently reported in vitro and in vivo IgE binding activity data, the UQCRB protein homolog identified in Der p was named Der p 24 and catalogued as such in the WHO/IUIS allergen database. The N-terminal 32-residue region of Der p 24 was bound by specific IgEs from HDM allergic sera in vitro and produced specific IgE antibodies in vivo, indicating that this region is an immunodominant IgE epitope of Der p 24 allergen.

Although the allergenic components of the globally important HDM species Der f and Der p [27] are highly homologous, differences in the sequences of their allergenic components can lead to differing IgE binding and reactivity sensitization mechanisms [12]. Hence, detailed knowledge about each major HDM allergen can inform the rational design of hypoallergenic vaccines and immunotherapy agents [28], particularly relative to agents produced from crude HDM extracts composed of a mixture of allergenic proteins and unrelated impurities that can produce highly variant effects, desensitization, and adverse secondary effects [29].

The epitopes of most HDM allergens have yet to be clearly resolved. The identification and analysis of immunodominant IgE epitopes of allergens, especially the major allergens, can help to elucidate allergen-induced sensitization mechanisms and provide information that can be used improving immunotherapy outcomes [30]. Prior to this study, the B cell epitopes of group 1, 2, 3, 7, 11, 13, 23 and 33 HDM allergens had been identified and IgE responses to HDMs had been shown to target serodominant epitopes, primarily, Der p 1, 2, and 23 components, with intermediate responses to Der p 4, 5, 7, and 21 components (titers proportional to serodominant specificities in 30–50% of subjects) [31].

Genetic recombinant hybrid proteins have become a favored immunotherapy approach [32]. Der p 1 and Der p 2 fragments in particular have emerged in candidate hypoallergenic molecules for HDM tolerance-development and vaccination approaches [7]. In this context, the identification of the immunodominant epitope of Der p 24 may facilitate the development of a broad-spectrum hypoallergenic vaccine for HDM allergy. The N-terminal 32-residue region of Der p 24 conjugated with OVA induced specific IgE production in vivo, indicating that it contains multi-type B cell epitopes, including a specific number of conformational and linear epitopes, capable of stimulating the innate immune pathways that trigger IgE production and allergy symptoms. Additionally, the BSA-Der p 24(1–32aa)-conjugate could enhance the proliferation of percentages of CD4 + T cells (Data not shown). It requires more experimental evidence to confirm which amino acid sites are critical for the T cell epitope of Der p 24. Further research is needed to elucidate the details of how immune pathways are activated by Der p 24 in vivo. Notwithstanding, the present data indicate that the novel allergen Der p 24, a UQCRB protein homolog, represents a good candidate for HDM allergy diagnosis and therapy applications owing to its specific allergenic sequences.

The ongoing discovery of HDM allergens may ultimately lead to a complete HDM allergen panel that can enable component-resolved diagnosis and patient-tailored therapies [33]. Component-resolved diagnostics makes use of defined allergen molecules to analyze IgE-mediated sensitization on a molecular level [34]. Der f 2 was shown to be the most frequently sensitized allergen among HDM-sensitized respiratory allergy and atopic dermatitis patients in a Korean study, wherein the combination of group 1 and 2 major allergens improved diagnostic sensitivity [35]. In a serological proteome analysis study, a group 11 HDM allergen was found to contain an HDM allergic serum IgE-recognized allergen component [36]. Interestingly, sensitization to Der p 10 was shown to result from seafood-cockroach cross-reactivity in a study with participants from coastal southern China [37]. Because we found that the IgE binding activity of the N-terminal 32-residue region of Der p 24 was stronger than that of linear IgE epitopes of Der p 1 or Der p 2, we investigated whether a peptide constituted by that region, namely Peptide-1–32aa, may be a component-resolved diagnostic biomarker for HDM allergy and may associate with particular allergic symptoms. Our negative IgE-ELISA results regarding associations with symptom manifestation may be due to the small sample size (N = 10 per symptom group). An analysis of a larger sample size may reveal symptom associations. Further studies are needed to analyze the relationship between group 24 HDM allergen sensitization and allergic disease expression.

Sensitization mechanisms have been elucidated for very few of the 38 recognized groups HDM allergens. Hence, questions remain regarding the allergenic mechanisms of HDM proteins, with potential mechanisms including native protein function effects and immunogenicity of unique sequences that form IgE B cell epitopes. The clinically important group 1 and 2 HDM allergens—which exhibit IgE-binding activity with sera from most patients with mite allergies [38]—were shown to induce Th2 immune responses through their protein functions. That is, Der p 1’s allergenicity is consequent to its native cysteine protease function [39] while Der p 2 facilitates Toll-like receptor 4 signaling [40]. On the contrary, we found that the immunodominant N-terminal 32-residue epitope of Der p 24 induced specific IgE production in vivo and bound serum IgEs from sensitized mice to promote mast cell degranulation without any apparent relation to the native Der p 24 UQCRB protein function. Hence, the particular mechanism of allergenicity appears to vary among HDM allergens.

## Conclusions

The presently reported experiments led to the identification of Der p 24 as a major HDM allergen with robust IgE binding activity. The immunodominant IgE epitope of Der p 24 was localized to the N-terminal 32-residue region, which produced high titers of specific IgE antibodies in vivo. The present demonstration that the Der p 24 N-terminal epitope exhibited stronger IgE binding activity than linear IgE epitopes of Der p 1 or Der p 2 suggests that it may be useful as a component resolved diagnostic maker for HDM allergic diseases. These findings have theoretical implications for the development of diagnostic and therapeutic reagents for HDM allergy.

## Data Availability

The datasets used and/or analysed during the current study are available from the corresponding author on reasonable request.
